# Association between secondhand smoke exposure and abnormal cervical cytology: A one-to-one matched case-control study

**DOI:** 10.18332/tid/99502

**Published:** 2018-11-23

**Authors:** Libin An, Xiaohua Zhou, Wentao Li, Yang Wang, Hongling Shi, Tienan Xie

**Affiliations:** 1 Dalian University, Dalian, China; 2 Jilin Science and Technology Vocational Technical College, Changchun, China; 3 Reproductive Center, First Hospital of Jilin University, Changchun, China

**Keywords:** secondhand smoke, case-control study, cervical cytology, liquid-based cytologic, one-to-one matched

## Abstract

**INTRODUCTION:**

The aim was to evaluate the association between secondhand smoke (SHS) exposure and abnormal cervical cytology among Chinese adult women.

**METHODS:**

A one-to-one matched case-control study was conducted with outpatients of the First Hospital of Jilin University between October 2013 to September 2016. In all, 228 cytologic confirmed new cases of abnormal cervical cytology and the equivalent number of age and ethnic matched controls were interviewed about SHS exposure and related factors.

**RESULTS:**

Although 78.3% of all the participants had been exposed to SHS (78.1% subjects vs 78.5% controls), there were no statistical significance of cervical cytological abnormalities and SHS exposure status (never, former, current exposure), exposure intensity in cigarettes per day (none, 1–9, 10–19, and ≥20), SHS exposure duration in years (none, 1–9, 10–19, and ≥20) and the Brinkman Index (BI) (none, 1–99, 100–399, ≥400) between the two groups. The univariate analysis results showed that there were statistical differences between subjects and controls in marital status, sexual frequency in past year, number of sexual partners, age at first intercourse, age at first delivery. The stratified Cox regression model only showed that the age at first sexual intercourse was associated with the cervical cytological abnormalities (OR=1.206, 95% CI: 1.104–1.319).

**CONCLUSIONS:**

Studies on the association between SHS exposure and cervical lesions have been equivocal. In this study, the SHS exposure could not be detected as an independent risk factor of abnormal cervical cytology among Chinese adult women.

## INTRODUCTION

Cervical cancer is ranked as the third most common genital system cancer among women^1,2^. As the most populated country in the world, China has the largest cervical cancer population. According to Cancer Statistics in China in 2015, 98900 new cervical cancer cases and 30500 deaths were reported, which makes it the most common cancer diagnosis of Chinese women’s genital system malignant tumors^3^.

The occurrence and development of cervical cancer are gradual cytological evolution processes. Human papilloma virus (HPV) infection and cervical intraepithelial neoplasia (CIN) can further develop into cervical cancer^4^. It takes 5–15 years for abnormal cervical cytology with precancerous lesions to develop into cervical cancer, hence the prevention and early treatment of cervical cancer can be achieved by screening abnormal cervical cytology^5^.

Cigarette smoking is one of the most critical risk factors for the development of cervical lesions^6-8^. Although the smoking rate of women is low in China (in Beijing for example, the rate is 2.8%), the prevalence of secondhand smoke exposure (SHS) for women is estimated at 45.7%^9^. The ‘Protect women from tobacco marketing and smoke’ report from WHO, 2010, pointed out that about 40% of males are smokers, while only 9% of females are smokers. About 0.43 million adults die every year due to secondhand smoke, of which 64% are women^10^.

Given that the prevalence of exposure to SHS among Chinese adult women, studying the association between exposure to SHS and cervical cytological abnormalities may be meaningful. We have conducted a one-to-one matched case-control study to investigate the association between SHS exposure and cervical cytological abnormalities in Chinese adult women.

## METHODS

### Participants

The study was conducted between October 2013 and September 2016. Participants were women older than 18 years of age who underwent the liquid-based cytologic test in the First Hospital of Jilin University. Pregnant women, women who were under the age of 18 years, or active smokers, were excluded from the study. Cervical smears were classified according to 2001 Bethesda System as within normal limits (WNL), atypical squamous cells of undetermined significance (ASCUS), low-grade squamous intraepithelial lesion (LSIL), high-grade squamous intraepithelial lesion (HSIL), squamous cell carcinoma (SCC). A total of 228 women with ASCUS, LSIL, HSIL, or SCC, were identified as cases.

Each study subject was assigned one candidate control. Each control was selected randomly from women with the liquid-based cytologic test result showing WNL at the same hospital and the same time and matched each respective subject with age (±2 years) and ethnic background.

This study was approved by the Ethics Committee of Dalian University. The ethical principles for medical research in the Declaration of Helsinki were strictly obeyed to protect the autonomy and privacy of the subjects. All subjects provided a consent form to participate in this study voluntarily.

### Survey method and content

Data were collected using a structured questionnaire that was designed by the researchers according to the studies of Ward KK^11^, Wu MT^12^, and others^13-15^. The questionnaire consisted of sociodemographic characteristics, family health history, menstrual history, sexual history, fertility history, and SHS exposure history. A one-to-one on-site investigation was carried out, and omissions were replenished timely to ensure the integrity of the information.

SHS exposure was evaluated at home and in the workplace, as well as during two stages of life, which were childhood (≤18 years) and adulthood (>18 years), respectively. SHS exposure was identified for non-smokers who had been exposed to SHS more than 15 minutes and at least one day per week. A series of detailed follow-up questions were asked, including SHS exposure intensity and duration. SHS exposure intensity in cigarettes per day (none, 1–9, 10–19, and ≥20), SHS exposure duration in years (none, 1–9, 10–19, and ≥20) and Brinkman Index (BI) (none, 1–99, 100–399, ≥400) calculated by multiplying the average number of cigarettes smoked per day by the smoking years^16^.

### Statistical analysis

The database was established by using EpiData3.02 software, and the statistical analysis was performed by using SPSS17.0 software. Descriptive data were reported as frequency, percentage, mean and standard deviation (M±SD). Comparison between categorical variables was examined by using chi-squared and two-related-samples tests. Paired-samples and independent-samples t-tests were performed to investigate the relationship of measurement data, such as the history of sex and fertility. Cox regression model test of survival analysis was used to assess the association between cases/controls and different amounts of SHS exposure. Variables included SHS exposure parameters and those with a significant difference in univariable analysis, namely marital status, sexual frequency in past year, number of sexual partners, age at first intercourse, age at first delivery.

## RESULTS

### Sociodemographic characteristics

The mean age of the cases was 42.27 (±11.9) years versus 42.25 (±11.9) of the controls, both with a range of 19–81 years. Of the 228 cases, 89 cases (39.0%) reported ASCUS, 54 cases (23.7%) LSIL, 60 cases (26.3%) HSIL, and 25 cases (11.0%) SCC. In this study, 58.6% (266/456) of women had an HPV-DNA detection in cervical samples (73.7% subjects vs 43.0% controls), and 58.3% (155/266) had an HPV infection (78.0% cases vs 24.5% controls, χ^2^=72.82 p<0.001).

The sociodemographic characteristics of the study population, stratified by cervical smear results, are shown in Table 1. The proportion of married in the controls was larger than that in the cases (χ^2^=4.0, p=0.046). In order to explore the reason, the number of sexual partners in past year between two marriages (Table 2) was compared. The result showed that single, divorced, separated, or widowed women had more sexual partners in past year than the married women (13.0% vs 3.1%).

**Table 1:** Distribution of sociodemographic characteristics

*Variable*	*Cases*	*Controls*		*χ*^2^	*p*
		*+*	*-*		
Education level	+	39	35	2.306	0.129
	-	50	104		
Marital status	+	169	17	4.000	0.046
	-	32	10		
Annual household income	+	77	60	0.736	0.391
	-	50	41		

Note: We divided education level into ≤ high school and > high school, marital status into married and others (including single, never married; divorced; separated, and widowed), annual household income into ≤2000 yuan and >2000 yuan. We defined > high school, married, and >2000 yuan as exposed (+), respectively.

**Table 2:** Number of sexual partners in the past year by marital status

*Marital status*	*Number of sexual partners in past year*		*χ2*	*p*
	*<2*	*≥2*		
	*n %*	*n %*		
Married	375 (96.9)	12 (3.1)	13.177	<0.001
Others	60 (87.0)	9 (13.0)		

### History of sex and fertility

Two-related-samples tests and paired-samples t-tests were performed, respectively, to investigate the relationship between sex and fertility histories of cases and controls (Tables 3 and 4). The results showed that cases had a higher number of sexual partners, more frequent sexual intercourse in the past year, younger age at first intercourse and younger age of delivery than controls.

**Table 3:** History of sex and fertility

*Variable*	*Cases*	*Controls*		*χ2*	*p*
		*+*	*-*		
Sexual frequency in the past year	+	4	24	5.939	0.015
	-	9	191		
Number of sexual partners	+	13	49	6.453	0.011
	-	26	140		

Note: We divided sexual frequency in past year into <3 times per week and ≥ 3 times per week, number of sexual partners into <2 and ≥2. We defined sexual frequency in the past year ≥ 3 times per week, and number of sexual partners ≥2 as exposed (+), respectively.

**Table 4:** History of sex and fertility

*Variable*	*Cases (M±SD)*	*Controls (M±SD)*	*t*	*p*
Age of menarche	14.79±4.95	14.85±3.58	0.144	0.886
Menstrual cycle	29.33±8.35	28.96±4.64	-0.594	0.553
Menstrual period	5.45±1.39	5.63±1.33	1.547	0.123
Age at 1st intercourse	22.04±2.90	23.29±2.61	4.934	0.000
Gravidity	2.48±1.34	2.40±1.34	-0.538	0.591
Abortion	1.07±1.12	0.97±1.16	-0.777	0.438
Parity	1.42±0.90	1.44±0.82	0.242	0.809
Spontaneous labor	1.30±0.99	1.31±0.95	0.072	0.943
Cesarean delivery	0.18±0.40	0.18±0.40	-0.142	0.887
Age at 1st delivery	24.22±3.33	25.34±3.14	3.299	0.001

M±SD: mean ± standard deviation.

### SHS exposure

In all, 78.3% of the participants were exposed to SHS (78.1% subjects vs 78.5% controls). The places of SHS exposure were primarily at home and in the workplace, with 70.0% and 50.2%, respectively (χ^2^=31.15, p<0.001). Of the cases, 33.8% reported exposure to SHS at home during adulthood versus 36.8% of controls (χ^2^=0.47, p=0.557). Exposure to SHS was compared between cases and controls according to their exposure status (never, former, current exposure), no difference between the two groups was found (Tables 5–7). To examine the relationship between exposure to SHS and abnormal cervical cytology in a multivariate context, a stratified Cox regression model was carried out. The result only showed that the age at first intercourse was associated with the cervical cytological abnormalities (OR=1.206, 95% CI: 1.104–1.319) (Table 8).

**Table 5:** SHS exposure intensity

*SHS exposure intensity (cigarettes/day)*		*Cases*	*Controls*	*Z*	*p*
		*n (%)*	*n (%)*		
The total exposure to SHS	0	50 (21.9)	48 (21.1)	-0.692	0.489
	1–9	39 (17.1)	50 (21.9)		
	10–19	30 (13.2)	31 (13.6)		
	≥20	109 (47.8)	99 (43.4)		
Exposure at home	0	67 (29.4)	70 (30.7)	-0.647	0.518
	1–9	44 (19.3)	50 (21.9)		
	10–19	35 (15.4)	32 (14.0)		
	≥20	82 (36.0)	76 (33.3)		
Current exposure at home	0	104 (45.6)	114 (50.0)	-1.393	0.164
	1–9	37 (16.2)	45 (19.7)		
	10–19	35 (15.4)	28 (12.3)		
	≥20	52 (22.8)	41 (18.0)		
Former exposure at home	0	153 (67.1)	144 (63.2)	-0.696	0.486
	1–9	21 (9.2)	26 (11.4)		
	10–19	16 (7.0)	21 (9.2)		
	≥20	38 (16.7)	37 (16.2)		
Exposure in the workplace*	0	81 (54.0)	70 (46.4)	-0.402	0.687
	1–9	16 (10.7)	38 (25.2)		
	10–19	11 (7.3)	6 (4.0)		
	≥20	42 (28.0)	37 (24.5)		

* The total number of participants working in a closed place was 301.

**Table 6:** SHS exposure duration

*SHS exposure duration (years)*		*Cases*	*Controls*	*Z*	*p*
		*n (%)*	*n (%)*		
The total exposure to SHS	0	50 (21.9)	49 (21.5)	-0.141	0.888
	1–9	31 (13.6)	30 (13.2)		
	10–19	37 (16.2)	43 (18.9)		
	≥20	110 (48.2)	106 (46.5)		
Exposure at home	0	67 (29.4)	71 (31.1)	-0.459	0.646
	1–9	22 (9.6)	23 (10.1)		
	10–19	39 (17.1)	38 (16.7)		
	≥20	100 (43.9)	96 (42.1)		
Current exposure at home	0	104 (45.6)	114 (50.0)	-0.653	0.514
	1–9	31 (13.6)	25 (11.0)		
	10–19	37 (16.2)	34 (14.9)		
	≥20	56 (24.6)	55 (24.1)		
Former exposure at home	0	153 (67.1)	145 (63.6)	-0.946	0.344
	1–9	4 (1.8)	10 (4.4)		
	10–19	27 (11.8)	16 (7.0)		
	≥20	44 (19.3)	57 (25.0)		
Exposure in the workplace	0	81 (54.0)	70 (46.4)	-1.452	0.146
	1–9	36 (24.0)	37 (24.5)		
	10–19	18 (12.0)	26 (17.2)		
	≥20	15 (10.0)	18 (11.9)		

**Table 7:** Brinkman Index (BI) of SHS exposure

*SHS exposure*		*Cases*	*Controls*	*Z*	*p*
		*n (%)*	*n (%)*		
The total exposure to SHS	0	50 (21.9)	49 (21.5)	-0.412	0.680
	1–99	34 (14.9)	38 (16.7)		
	100–399	57 (25.0)	45 (19.7)		
	≥400	87 (38.2)	96 (42.1)		
Exposure at home	0	67 (29.4)	71 (31.1)	-0.069	0.945
	1–99	36 (15.8)	40 (17.5)		
	100–399	61 (26.8)	42 (18.4)		
	≥400	64 (28.1)	75 (32.9)		
Current exposure at home	0	104 (45.6)	114 (50.0)	-0.994	0.320
	1–99	37 (16.2)	43 (18.9)		
	100–399	60 (26.3)	40 (17.5)		
	≥400	27 (11.8)	31 (13.6)		
Former exposure at home	0	153 (67.1)	145 (63.6)	-0.801	0.423
	1–99	17 (7.5)	18 (7.9)		
	100–399	28 (12.3)	31 (13.6)		
	≥400	30 (13.2)	34 (14.9)		
Exposure in the workplace	0	81 (54.0)	70 (46.4)	-0.865	0.387
	1–99	27 (18.0)	44 (29.1)		
	100–399	27 (18.0)	16 (10.6)		
	≥400	15 (10.0)	21 (13.9)		

**Table 8:** The stratified Cox regression analysis of SHS and abnormal cervical cytology

*Variable*	*B*	*SE*	*Wald*	*p*	*Exp(B)*	*95% CI*
Age at 1st intercourse	0.188	0.045	17.018	<0.001	1.206	1.104–1.319

## DISCUSSION

The association between SHS exposure and cervical lesions is uncertain. Some research results showed that cervical lesions were associated with SHS exposure^11,17-21^, while others failed to show an association^22,23^. Possible risk factors for cervical lesions including menstrual history, sexual history, fertility history and SHS exposure history were investigated in this study. Because the incidence of abnormal cervical cytology is different for people at different age and ethnicity^24-27^, we matched with age and ethnicity between cases and controls.

This investigation was carried out in northeast China, where cigarette smoking is prevalent. Through the analysis of the results, we did not find any association between SHS exposure and abnormal cervical cytology, even though SHS exposure was evaluated at home and in the workplace, as well as during two stages of life, childhood (≤18 years) and adulthood (>18 years), respectively. This finding is not consistent with other studies^11,17-21^. A possible explanation is the cumulative effects of SHS exposure. Some soluble compounds such as nicotine and cotinine may have a direct transforming effect on the cervical epithelium, which affects the cervical metaplasia and leads to malignant transformation^18,28^. Harmful substances such as nicotine in cigarettes could be absorbed by the body, and distributed to the whole body with the blood circulation, then discharged gradually. Cervix is a small part of the body and the accumulation of nicotine is far less than in the lung, which is rich in blood.

For this reason, we hypothesize that the duration of exposure to nicotine and other harmful substances must exist for a long time before causing cervical lesions. A meta-analysis^29^, on the relationship between smoking and lung cancer, showed smoking for more than 40 years could increase the incidence of lung cancer significantly (OR=10.77). In this study, the average SHS exposure time was 17.9 years, hence the cumulative effect of SHS exposure may not be obvious.

SHS exposure intensity was by self-report of the participants. The strong subjectivity may generate recall bias. In future work, the levels of nicotine and other harmful substances of cigarettes should be ascertained by blood samples or cervical discharge.

The univariate analysis results showed that multiple sexual partners, high-frequency sexual intercourse, and sexual intercourse at a younger age were risk factors for abnormal cervical cytology. These findings are consistent with other studies^11,12,30,31^. Multiple sexual partners could easily cause the cross-infection of the reproductive organs and may increase the risk of cervical disease and lesions. High-frequency sexual intercourse and sexual intercourse at a younger age could injure the cervical glandular mucous that can cause inflammatory changes. Wu MT^12^ considered that with increasing pregnancy frequency, the incidence of CINII and more severe disease would increase. However, in this study, only delivery at a younger age was found to be a risk factor of abnormal cervical cytology. The reason is unknown, but we speculate that the injury of the cervical tissue structure caused by delivery^32^ at a younger age might be the reason. The stratified Cox regression model only showed that the age of first sexual intercourse was associated with the cervical cytological abnormalities. First sexual intercourse at an older age was a protective factor for cervical lesions.

The major limitation of this study was lack of information about HPV infection. HPV infection has a well-documented association with the risk of cervical neoplasm^33,34^, but not all the participants had the results of HPV-DNA in this study.

## CONCLUSIONS

Although no association between SHS exposure and abnormal cervical cytology was found, the effects of SHS exposure on women cannot be ignored. Public awareness of the dangers of SHS exposure should be enhanced. In addition, it is necessary that cervical screening should be performed for married women every 3 to 5 years, depending on age and screening modality (Pap test only or combined with the HPV-DNA test, referred to as ‘co-testing’ )^35^, in order to diagnose and intervene early, and to reduce the incidence and mortality of cervical cancer.
